# Uric Acid Levels in Normotensive Children of Hypertensive Parents

**DOI:** 10.1155/2015/747082

**Published:** 2015-08-24

**Authors:** Ali Yildirim, Fatma Keles, Pelin Kosger, Gokmen Ozdemir, Birsen Ucar, Zubeyir Kilic

**Affiliations:** Department of Pediatric Cardiology, Medical Faculty, Eskisehir Osmangazi University, 26480 Eskisehir, Turkey

## Abstract

This study evaluated uric acid concentrations in normotensive children of parents with hypertension. Eighty normotensive children from families with and without a history of essential hypertension were included. Concentrations of lipid parameters and uric acid were compared. Demographic and anthropometric characteristics were similar in the groups. Systolic and diastolic blood pressure were higher in the normotensive children of parents with hypertension without statistically significant difference (*P* > 0.05). Uric acid concentrations were higher in the normotensive children of parents with hypertension (4.61 versus 3.57 mg/dL, *P* < 0.01). Total cholesterol and triglyceride concentrations were similar in the two groups. Systolic and diastolic blood pressure were significantly higher in control children aged >10 years (*P* < 0.01). Uric acid levels were significantly higher in all children with more pronounced difference after age 10 of years (*P* < 0.001). Positive correlations were found between the level of serum uric acid and age, body weight, body mass index, and systolic and diastolic blood pressure in the normotensive children of parents. The higher uric acid levels in the normotensive children of hypertensive parents suggest that uric acid may be a predeterminant of hypertension. Monitoring of uric acid levels in these children may allow for prevention or earlier treatment of future hypertension.

## 1. Introduction

Hyperuricemia has been associated with various diseases, including gout, hypertension, atherosclerosis, cardiovascular events, the metabolic syndrome, and renal failure [1]. Studies in experimental animals have shown that increased serum levels of uric acid are related to cardiovascular risk and hypertensive cardiovascular damage [2–8]. High levels of serum uric acid in rats increased blood pressure and blood pressure decreased to normal level with treatment of hyperuricemia [9]. Moreover, hyperuricemia may lead to renal tubulopathy by decreasing the synthesis of nitric oxide [10–12]. Similarly, clinical studies have shown relationships between high levels of serum uric acid concentrations and increased blood pressure [13, 14]. For example, the incidence of hypertension was high in adolescents with high level serum uric acid, while treatment with allopurinol reduced both serum uric acid levels and blood pressure.

Several studies have investigated the relationship between hyperuricemia and hypertension in children [14–17]. Increased levels of uric acid in children were associated with increases in systolic and diastolic blood pressure, with increased uric acid being an independent risk factor for hypertension [18].

Essential hypertension is frequently observed in adults throughout the world, and its incidence is also increasing in children and adolescents [19]. Family history of essential hypertension has been shown to be a significant risk factor for essential hypertension in children [20]. Children of hypertensive parents are significantly more likely to have higher blood pressure during their adolescent years than the children of normotensive parents [19, 21]. The probability of hypertension developing in a child has been reported to be 28% if one parent has hypertension and 41% if both parents have hypertension [19].

Although several studies have assessed the relationship between essential hypertension and hyperuricemia in children, none to date has evaluated hyperuricemia in the normotensive children of hypertensive parents. This study therefore compared serum uric acid concentrations in the normotensive children of normotensive and hypertensive parents and the relationship between hyperuricemia and blood pressure in these children.

## 2. Materials and Methods

Of the 80 children included in the study, 36 were normotensive children from families with a history of essential hypertension, while 44 were normotensive children from families with no history of hypertension. All of these children had visited the Pediatric Cardiology Department of the Eskisehir Osmangazi University Faculty of Medicine (Eskisehir, Turkey) for murmurs, palpitations, or chest pains. The detailed medical history, background, and family history of the children were investigated. Children with a history of a chronic illness, prematurity, anemia, or use of medication for anemia, congenital heart disease, chronic renal failure, diabetes, hyperlipidemia, or obesity were excluded. Parents of the participants were provided with information on the nature and content of the study, and all provided written informed consent. The study protocol was approved by the Ethics Committee of Eskisehir Osmangazi University Medical School.

The children were divided into two groups. The study group consisted of children with a hypertensive mother and/or father (*n* = 36), while the control group consisted of children with no familial history of hypertension (*n* = 44). Parents of children in the study group had been diagnosed with essential hypertension and regularly used antihypertensive medications, with none having a history of additional illnesses causing secondary hypertension. Children of parents with a history of cardiovascular diseases, chronic renal failure, diabetes, hyperlipidemia, obesity, using the drug, and smoking were excluded from the study.

Weight and height were measured while the child was in the upright position without wearing shoes, and body mass index (BMI) was calculated as weight (kg)/height (m^2^). Blood pressure was measured three times with a mercury sphygmomanometer and appropriate armband while the subject was in a sitting position after 10 minutes of rest, with the mean of the three measurements recorded. After Korotkoff sounds were heard in the brachial artery, the armband was inflated 20 mmHg above the point at which brachial pressure was not apparent and the pressure was decreased by releasing air at 2-3 mmHg/sec. Korotkoff phase 1 was considered the systolic pressure, and Korotkoff phases 4 and 5 were considered the diastolic pressure for children aged <12 and >12 years, respectively.

Venous blood samples were taken from the antecubital vein of each subject after a 12-hour fast. After clotting, the samples were centrifuged at 4000 rpm for 5 minutes; the serum samples were removed and stored at −80°C until being analyzed. Serum concentrations of total cholesterol, total triglycerides, high-density lipoprotein-cholesterol (HDL-C), low-density lipoprotein-cholesterol (LDL-C), and uric acid were measured enzymatically with a Roche modular device.

### 2.1. Statistical Analysis

IBM SPSS Statistics 20 software was used for all statistical analyses. The normal distribution of variables was evaluated using the Shapiro-Wilk test. Normally distributed variables were reported as mean ± standard deviation (SD) and compared using Student's *t*. The Mann-Whitney *U* test was performed for variables with nonnormal distributions, and median and percentile values (25–75%) were obtained. The numerical variables of three age groups were compared by ANOVA (one-way analysis of variance) test and pairwise comparisons were performed by post hoc test. To evaluate the relationships between the variables, a Spearman correlation analysis was performed. Statistical significance was defined as *P* < 0.05.

## 3. Results

The study cohort consisted of 36 normotensive children, 15 males (37.5%) and 21 females (62.5%), aged 7 to 20 years (mean age, 12.4 years), each of whom had at least one hypertensive parent (Group 1), and 44 normotensive children, 17 males (33.3%) and 27 females (66.7%), aged 6 to 20 years (mean age, 12.7 years), none of whose parents had any health problems, including hypertension (Group 2). Their anthropometric measurements, blood pressure, and laboratory values are shown in Table 1. The mean weights of the children in Groups 1 and 2 were 59.2 kg and 57.3 kg, respectively; their mean heights were 166.5 cm and 164.8 cm, respectively; and their mean BMIs were 21.2 kg/m^2^ and 21.0 kg/m^2^. There were no statistically significant differences (*P* > 0.05) between the study and control groups in age, gender, weight, height, or BMI. Children in Group 1 had a mean systolic blood pressure (SBP) of 113 mmHg and a mean diastolic blood pressure (DBP) of 69 mmHg, whereas children in Group 2 had a mean SBP of 108 mmHg and a mean DBP of 65 mmHg. Although both SBP and DBP were higher in Group 1 than in Group 2, these differences were not statistically significant (*P* > 0.05). Serum uric acid levels, however, were significantly higher in Group 1 than in Group 2 children (4.31 mg/dL versus 3.37 mg/dL, *P* < 0.01). Concentrations of total cholesterol, HDL-C, LDL-C, and total triglycerides, however, were similar in the two groups (*P* > 0.05 each).

In order to detect the elevation period of uric acid level and to determine whether an age-related change in variables is present, patients were divided into 3 groups according to their age. Thus, there were 9 patients < 10 years of age, 12 patients between 10 and 15 years of age, and 15 patients over 15 years of age. Control group consisted of 13 subjects under 10 years of age, 15 subjects between 10 and 15 years of age, and 16 subjects over 15 years of age. There were no significant differences regarding SBP, DBP, total cholesterol, HDL-C, LDL-C, and triglycerides between the study and control groups based on age (*P* < 0.05) (Table 2). Uric acid concentrations were significantly higher in Group 1 than in Group 2 at all ages, but this difference was especially pronounced in subjects aged >10 years (*P* < 0.001). The differences between variables according to different age groups are shown in Table 3. For all variables, a statistical difference was present according to age groups.

Correlations between uric acid and other variables are shown in Table 4. Uric acid levels showed positive correlations with age, body weight, BMI, SBP, DBP, and triglyceride concentrations in children from Group 1. Uric acid concentrations, however, did not significantly correlate with total cholesterol, HDL-C, and LDL-C concentrations in these subjects (*P* > 0.05). While there was a correlation between uric acid, DBP, and triglycerides in Group 2, no correlations with other variables were observed (*P* > 0.05).

## 4. Discussion

This study found no significant difference in SBP and DBP between the normotensive children of normotensive and hypertensive parents. Previous studies have shown a clear correlation between high blood pressure and a family history of hypertension [22–27], especially after adolescence [22]. Studies of prepubertal and pubertal children of normotensive and hypertensive parents showed no significant differences in blood pressure [28, 29]. For example, one study reported blood pressure was similar in the 18–22-year-old children of hypertensive and normotensive parents but aortic stiffness parameters were higher in the former group [29]. In our previous study we found that an increase in the carotid intima-media thickness in children of hypertensive parents may indicate subclinical atherosclerosis [30]. Children of hypertensive parents may first experience hormonal and biochemical changes, followed by the development of damage to the endothelial and vascular structures and subsequent cardiac compensation, with hypertension only appearing at latter stages. Moreover, in this study the hypertensive and prehypertensive children of hypertensive parents were not included and the age range of the participants was narrow, perhaps explaining, at least in part, the similar SBP and DBP in the study and control groups.

Despite the uric acid levels of normotensive children of hypertensive parents being within normal boundaries, they were significantly higher than in normotensive children of normotensive parents. In order to detect the elevation period of uric acid level and to determine whether an age-related change in variables is present, patients were divided into 3 groups according to their age. Patients in all age groups had increased uric acid levels as compared to controls, with a more marked elevation in uric acid among those over 15 years of age. Many previous studies have reported a relationship between hyperuricemia and hypertension. For example, a study of 200 children found that 73% of those with medium and severe levels of hypertension had uric acid levels >7 mg/dL, compared with 49% of those with low levels of hypertension [15]. Hyperuricemia was observed in very young children, as well as being an early indicator of hypertension. A 13-year study of children born in Budapest, Hungary, found that the development of hypertension was associated with increased heart rate, early sexual maturity, and hyperuricemia [16]. Another study found that the development of hypertension over 10 years was associated with high levels of serum uric acid, independent of the glomerular filtration rate, alcohol consumption, smoking, and diabetes [29]. Uric acid and renin levels were found to be higher in adolescents aged 13–18 years with essential hypertension than in an age- and gender-matched normotensive control group [17]. Moreover, renin levels were normal in hypertensive individuals with normal levels of serum uric acid but higher in hypertensive individuals with high level of serum uric acid. Hyperuricemia was found to increase the synthesis of nitric oxide, increasing the release of inflammatory mediators and renin angiotensin levels and resulting in endothelial dysfunction, atherosclerosis, and hypertension [31–33]. In its early stages, hyperuricemia leads to vasoconstriction, whereas, in more advanced stages, hyperuricemia causes hypertrophy of vascular smooth muscles, increasing blood pressure. Initially, increased blood pressure responds well to treatment, but the response is reduced at more advanced stages [34]. Pharmacologic induction of hyperuricemia in mice resulted in hypertension after three weeks, whereas treatment with uricosuric agents reduced hypertension in these animals, indicating that hyperuricemia is an independent risk factor for hypertension [12]. A placebo-controlled trial in 60 prehypertensive and obese children found that treatment with allopurinol or probenecid reduced SBP by 10 mmHg and DBP by 9 mmHg, indicating that treatment of hyperuricemia can reduce hypertension [35]. Despite many studies of the relationship between hypertension and uric acid, none had previously investigated uric acid levels in the normotensive children of hypertensive parents. Our finding that serum uric acid levels were higher in the normotensive children of hypertensive parents than normotensive parents suggests that uric acid increases first, prior to the development of hypertension, with hypertension developing later. The higher uric acid levels in the normotensive children of hypertensive parents suggest that uric acid may be a predeterminant of hypertension. Monitoring of uric acid levels in these children may allow for prevention or earlier treatment of future hypertension. The increase in uric acid was observed at very young ages, 5–10 years, and may be followed by the development of endothelial damage and vascular stiffness and eventually the appearance of hypertension.

We observed that uric acid levels were positively correlated with SBP, DBP, BMI, age, and triglyceride concentrations. Serum uric acid levels were found to be higher in patients with essential hypertention than with secondary hypertension [14]. Serum uric acid levels were linearly correlated with SBP and DBP, with a 1 mg/mL increase in uric acid resulting in a 14 mmHg increase in SBP and a 7 mmHg increase in DBP [14]. In addition, a meta-analysis found that a 1 mg/dL increase in serum uric acid levels increased the incidence of hypertension by 13% [36]. That analysis reported a linear relationship between blood pressure and uric acid, rather than a cutoff point.

In conclusion, this study found that the levels of serum uric acid were higher at all ages in the normotensive children of hypertensive than normotensive parents. The increased levels of serum uric acid in the former may be a predeterminant of hypertension. Long term studies in large numbers of children of hypertensive parents are needed to assess the relationship between blood pressure and serum uric acid levels and the impact of diet on both levels in such children. Monitoring of uric acid may reveal early stages of hypertension in these children, allowing the implementation of measures to prevent hypertension.

## Figures and Tables

**Table 1:** Demographic characteristics of normotensive children of hypertensive parents (Group 1) and normotensive parents (Group 2).

	Group 1 (*n* = 36)	Group 2 (*n* = 44)	*P*
Age, yrs^#^	12.4 ± 3.9	12.7 ± 4.1	0.973
Female^$^ *n*	21	27	0.891
Male *n*	15	17	0.765
Weight, kg^+^	59.2 ± 12.4	57.3 ± 13.6	0.932
Height, cm^+^	166.5 ± 19.0	164.8 ± 17.1	0.758
BMI, kg/m^2+^	21.2 ± 3.2	21.0 ± 2.8	0.194
SBP, mmHg^#^	113.2 ± 9.0	108 ± 8.2	0.345
DBP, mmHg^#^	69.0 ± 8.2	65.1 ± 7.0	0.621
Triglycerides, mg/dL^#^	80.7 ± 31.4	77.4 ± 30.6	0.106
HDL-cholesterol, mg/dL^#^	51.8 ± 10.4	55.7 ± 13.7	0.325
LDL-cholesterol, mg/dL^#^	86.8 ± 22.8	86.2 ± 23.4	0.156
Total cholesterol, mg/dL^+^	148.7 ± 25.4	152.3 ± 30.1	0.765
Uric acid, mg/dL^+^	4.31 ± 1.12	3.37 ± 0.89	0.007

BMI: body mass index; DBP: diastolic blood pressure; HDL: high-density lipoprotein; LDL: low-density lipoprotein; SBP: systolic blood pressure. All results reported as mean ± SD; ^#^Student's *t*; ^$^Pearson chi-square test; ^+^Mann-Whitney *U* test.

**Table 2:** Blood pressure, lipid parameters, and uric acid levels by age in normotensive children of hypertensive parents (Group 1) and normotensive parents (Group 2).

	Group 1 Age < 10 yrs	Group 2 Age < 10 yrs	Group 1 Age 10–15 yrs	Group 2 Age 10–15 yrs	Group 1 Age > 15 yrs	Group 2 Age > 15 yrs
SBP, mmHg	102 ± 6.5	99 ± 5.6	111.6 ± 8.3	108 ± 5.9	115 ± 4.9	112 ± 6.0
DBP, mmHg	60.5 ± 7.2	62.4 ± 4.6	69.0 ± 7.3	65.8 ± 5.9	71.0 ± 6.0	72.1 ± 6.4
Triglyceride, mg/dL	69.9 ± 21.6	77.0 ± 28.0	87.3 ± 30.6	83.8 ± 31.6	81.7 ± 37.3	72.0 ± 32.6
HDL-cholesterol, mg/dL	50.9 ± 10.4	57.2 ± 15.1	52.3 ± 10.7	55.5 ± 15.1	52.4 ± 10.4	55.0 ± 12.0
LDL-cholesterol, mg/dL	84.8 ± 18.6	91.4 ± 15.4	92.5 ± 28.6	86.3 ± 24.3	81.8 ± 17.0	82.6 ± 21.1
Total cholesterol, mg/dL	144.3 ± 22.8	159.1 ± 18.5	155.4 ± 31.3	153.3 ± 31.4	144.2 ± 17.1	147.0 ± 34.7
Uric acid, mg/dL	3.68 ± 0.75^≠^	3.20 ± 0.56	4.36 ± 0.99^≠^	3.03 ± 0.81	4.61 ± 1.34^≠≠^	3.57 ± 1.07

DBP: diastolic blood pressure; HDL: high-density lipoprotein; LDL: low-density lipoprotein; SBP: systolic blood pressure. All results reported as mean ± SD; ^≠^ *P* < 0.05, ^≠≠^ *P* < 0.01.

**Table 3:** Comparison of variables according to age groups.

Variables	Group 1				Group 2			
	*P*	*P*_1-2_	*P*_1–3_	*P*_2-3_	*P*	*P*_1-2_	*P*_1–3_	*P*_2-3_
SBP, mmHg	**<0.001**	**<0.001**	**<0.001**	**0.008**	**<0.001**	**<0.001**	**<0.001**	**0.002**
DBP, mmHg	**<0.001**	**<0.001**	**<0.001**	0.363	**<0.001**	**0.013**	**<0.001**	**<0.001**
Triglyceride, mg/dL	**<0.001**	**<0.001**	**<0.001**	**<0.001**	**<0.001**	**0.001**	**0.001**	**<0.001**
HDL-cholesterol, mg/dL	**0.004**	**0.003**	0.072	0.491	**0.012**	**0.022**	0.055	0.999
LDL-cholesterol, mg/dL	**<0.001**	**<0.001**	0.066	**<0.001**	**<0.001**	**<0.001**	**<0.001**	**<0.001**
Total cholesterol, mg/dL	**<0.001**	**<0.001**	0.999	**<0.001**	**<0.001**	**<0.001**	**<0.001**	**<0.001**
Uric acid, mg/dL	**<0.001**	**0.015**	**<0.001**	**0.006**	**0.036**	0.514	0.280	**0.032**

*P*: ANOVA test; pairwise comparisons were performed by post hoc test. DBP: diastolic blood pressure; HDL: high-density lipoprotein; LDL: low-density lipoprotein; SBP: systolic blood pressure. Comparison of groups: *P*_1-2_: age < 10 yrs and age 10–15 yrs; *P*_1–3_: age < 10 yrs and age > 15 yrs; *P*_2-3_: age 10–15 yrs and age > 15 yrs.

**Table 4:** Correlation between uric acid levels and other variables.

	Uric acid, mg/dL	Age, yrs	Height, cm	Weight, kg	BMI, kg/m^2^	SBP, mmHg	DBP, mmHg	TG, mg/dL	HDL-chol., mg/dL	LDL-chol., mg/dL	Total chol., mg/dL
Group 1 (*n* = 24)	*r*	0.701^**∗∗**^	0.569^**∗∗**^	0.436^**∗**^	0.518^**∗**^	0.663^**∗∗**^	0.552^**∗∗**^	0.478^**∗**^	0.241	−0.379	−0.062
	*P*	**<0.001**	**0.004**	**0.033**	**0.004**	**<0.001**	**0.005**	**0.007**	0.256	0.068	0.773

Group 2 (*n* = 27)	*r*	0.094	0.060	0.048	0.204	0.100	0.445^**∗**^	−0.408^**∗**^	0.088	−0.249	−0.120
	*P*	0.640	0.767	0.810	0.307	0.621	**0.020**	**0.034**	0.662	0.210	0.551

All correlations were calculated by Spearman correlation test; DBP: diastolic blood pressure; HDL: high-density lipoprotein; LDL: low-density lipoprotein; SBP: systolic blood pressure.

^*∗*^ *P* < 0.05, ^*∗∗*^ *P* < 0.01.
